# Analysis of N6-Methyladenosine Methylation Modification in Fructose-Induced Non-Alcoholic Fatty Liver Disease

**DOI:** 10.3389/fendo.2021.780617

**Published:** 2021-12-07

**Authors:** Yunchen Luo, Zhijian Zhang, Liping Xiang, Bing Zhou, Xuejiao Wang, Yi Lin, Xiaoying Ding, Fang Liu, Yan Lu, Yongde Peng

**Affiliations:** ^1^ Department of Endocrinology and Metabolism, Shanghai General Hospital, Shanghai Jiao Tong University School of Medicine, Shanghai, China; ^2^ Department of Endocrinology and Metabolism, Zhongshan Hospital, Fudan University, Shanghai, China

**Keywords:** fructose, lipid metabolism, non-alcoholic fatty liver disease, N6-methyladenosine, RNA methylation

## Abstract

Improvements in living standards have led to non-alcoholic fatty liver disease (NAFLD), one of the most common chronic liver diseases worldwide. Recent studies have shown that N6-methyladenosine (m6A), a type of RNA modification, is strongly associated with many important biological processes. However, the relationship between m6A methylation modifications and NAFLD remains poorly understood. In the present study, through methylated RNA immunoprecipitation sequencing and RNA transcriptome sequencing in high fructose diet-induced NAFLD mice, we found that hypermethylation-encoding genes were mainly enriched in lipid metabolism processes. We identified 266 overlapping and differentially expressed genes (DEGs) that changed at both the mRNA expression level and m6A modification level. Among them, 193 genes displayed increased expression and m6A modification, indicating that m6A RNA modifications tend to be positively correlated with NAFLD. We further compared the high fructose diet-induced NAFLD mouse model with leptin receptor-deficient mice and found that DEGs enriched in the lipid metabolism pathway were up-regulated in both groups. In contrast, DEGs associated with the immune inflammatory response were up-regulated in the high fructose diet group, but down-regulated in leptin receptor-deficient mice. Taken together, our results demonstrate that m6A methylation modifications may play an important role in the development of NAFLD.

## Introduction

Rapid changes in lifestyles have led to an increased prevalence of metabolic syndrome. Indeed, the manifestation of metabolic syndrome in the liver, non-alcoholic fatty liver disease (NAFLD), has become the most common chronic liver disease worldwide. NAFLD includes a series of clinical features, from simple liver steatosis and non-alcoholic steatohepatitis (NASH) to liver fibrosis, cirrhosis and hepatocellular carcinoma (1). The current global prevalence of NAFLD is approximately 25.2%, and is expected to increase in the future. Meanwhile, among NAFLD patients, the prevalence of NASH diagnosed by liver biopsy with clinical signs and symptoms is as high as 59%, while the prevalence of asymptomatic NASH is approximately 7%–30% (2).

The main manifestation of NAFLD is the excessive deposition of triglycerides (TGs) in the liver. Steatosis occurs when the input rate of fatty acid synthesis (*de novo* lipogenesis (DNL) and fatty acid uptake) exceeds the output rate of fatty acids (fatty acid oxidation and very low-density lipoprotein transport) (3). DNL is a complex metabolic reaction that enables the liver to synthesize new fatty acids from acetyl-CoA. In this process, the two key transcription factors are sterol regulatory element binding protein 1c (SREBP-1c) and carbohydrate response element binding protein (ChREBP). Most of the fatty acid synthesis rate-limiting enzymes, including fatty acid synthase (FASN), acetyl CoA carboxylase 1 (ACC1) and stearoyl-CoAdesaturase1 (SCD1), are transcriptionally activated by these two transcription factors (4). Fatty acid uptake by the liver is dependent on fatty acid transporters, including fatty acid transport protein and cluster of differentiation 36 (CD36) (5). In contrast, fatty acid oxidation is mainly regulated by the peroxisome proliferator-activated receptor (PPAR) alpha and its downstream key enzymes, including medium-chain acyl-CoA dehydrogenase, acyl-CoA oxidase and carnitine palmitoyl transferase (6). Very low-density lipoprotein transport is a complex lipoprotein particle that converts insoluble TG into a water-soluble form, which is then exported from the liver and transported to peripheral tissues (3).

Many risk factors have been linked to the pathogenesis of NAFLD. Fructose intake is considered to be a key factor in the development of NAFLD and has been reported to induce liver lipid deposition in humans (7) and experimental animals (8). Fructose is both a substrate and activator of DNL through the activation of several key transcription factors, such as SREBP-1c and ChREBP (9). Fructose can also induce mitochondrial dysfunction and promote oxidative stress through the production of reactive oxygen species and lipotoxicity associated with liver lipid metabolism disorders (10). Furthermore, under the continuous stimulation of fructose, endoplasmic reticulum stress promotes the activation of transcription factors such as Janus kinase and Nuclear Factor κB, which play an important role in the progression of NAFLD (11). However, the molecular mechanisms underlying high fructose intake-induced NAFLD remain unclear.

N6-methyladenosine (m6A), one of the most abundant modifications in mammalian mRNA, regulates various aspects of RNA metabolism including RNA maturation, splicing, transport, folding, translation, and decay (12–15). m6A modification is a dynamic and reversible process, which is regulated by m6A methyltransferases (also known as “writers”) and demethylases (also known as “erasers”). The typical m6A methyltransferase complex is mainly composed of methyltransferase 3 (METTL3), METTL14 and Wilm’s tumor 1-associated protein (16). m6A methylation modifications can be removed by demethylases, which mainly include obesity-related proteins such as fat mass and obesity associated protein and ALKB homolog 5 (ALKBH5) (17, 18). In addition, m6A is bound by m6A recognition proteins (also known as “readers”), including the YT521-B homology (YTH) domain family, insulin-like growth factor 2 mRNA-binding proteins and heterogeneous nuclear ribonucleoproteins (19). Recent studies have shown that m6A methylation is involved in many important biological events and human diseases, such as cancer pathogenesis (19), hematopoiesis dysfunction (20), immune system responses (21), cardiovascular diseases (22) and neurological diseases (23). In addition, our recent study found that RNA methylation could regulate hepatic TG metabolism and participate in the obesity-associated NAFLD (24). However, whether RNA methylation is involved in NAFLD caused by high fructose consumption is still unclear.

In the present study, through methylated RNA immunoprecipitation sequencing (MeRIP-Seq) and RNA transcriptome sequencing (RNA-Seq) analysis, we demonstrate that m6A RNA methylation plays an important role in the regulation of high-fructose diet (HFrD)-induced NAFLD. By comparing leptin receptor-deficient (*db/db*) mice and HFrD mice, we further show that the differentially expressed genes (DEGs) enriched in the lipid metabolism pathway are up-regulated in both groups, while DEGs enriched in the immune inflammatory response are up-regulated in HFrD mice and down-regulated in *db/db* mice.

## Materials and Methods

### Animal Experiments and Sample Collection

Male C57BL/6J mice aged 10-11 weeks were purchased from the Shanghai Laboratory Animal Company (SLAC, Shanghai, China). Eight-week-old *db/db* mice were purchased from Nanjing Biomedical Research Institute of Nanjing University (NBRI, Nanjing, China). All mice were kept at a temperature of 21 ± 1°C, a humidity of 55% ± 10%, and in a 12-hour light/dark cycle. HFrD containing 10% kcal from fat, 70% kcal from carbohydrate, and 20% kcal from protein were purchased from Research Diets (D08040107, Research Diets, USA). C57BL/6J mice were fed normal diet (ND) or HFrD for two weeks, then liver tissues were collected and stored in liquid nitrogen or fixed for pathological staining. The paraffin-embedded tissue is used for sectioning and H&E staining. Frozen sections were used for oil red O staining. Blood samples were collected and centrifuged at 3000×g for 15 minutes at 4°C to collect plasma.

### Liver Triacylglycerol Assay and Biochemical Analysis

The plasma levels of TG and total cholesterol (TC), alanine aminotransferase (ALT), and aspartate aminotransferase (AST) in the liver were determined using a commercial kit (Kehua, Shanghai, China) according to the manufacturer’s instructions. For liver triacylglycerol determination, liver tissue (~100 mg) was homogenized in 1 ml 5% NP40. The homogenized mixture was then heated in a water bath for 2–5 minutes at 95°C, and centrifuged at 15000×g for 15 minutes. The supernatant was collected and the triglyceride quantification kit (BioVision, Milpitas, USA) was used to determine the TG concentration.

### RNA Isolation and Real-Time PCR Analysis

Total RNA was extracted from the liver using TRIzol (Invitrogen, CA, USA) according to the manufacturer’s instructions. rRNA was removed from the total RNA (1 μg) using the Ribo-Zero rRNA Removal Kit (Illumina, San Diego, CA, USA) following the manufacturer’s instructions. RNA was reverse transcribed to cDNA using a reverse transcription assay kit (Life Technologies, Carlsbad, CA, USA). mRNA expression was determined using the SYBR Green PCR Mix (Thermo Fisher Scientific, Waltham, MA, USA). Gene expression was normalized to β-actin expression, and data were analyzed using the 2^−ΔΔCt^ method.

### Protein Extraction and Western Blots

Tissues are homogenized in Radio-Immunoprecipitation Assay buffer containing a mixture of protease and phosphatase inhibitors. The protein concentration was detected by the BCA protein assay kit (Thermo Fisher Scientific, Waltham, MA, USA), followed by SDS-PAGE electrophoresis to separate the protein and transfer it to a PVDF membrane (Millipore, Bedford, MA, USA). The membranes were incubated with the primary antibody (anti-FASN antibody (CST, #3180), anti-ACC1 antibody [CST, #4190), anti-GAPDH antibody (Santa Cruz, sc-32233)]. Use Image J for quantification of immunoblotting.

### MeRIP-m6A-Seq, RNA-Seq and Data Analysis

The MeRIP-m6A-Seq and RNA-Seq service was provided by Cloudseq Biotech Inc. (Shanghai, China). Briefly, m6A RNA immunoprecipitation (IP) was performed with the GenSeq™ m6A-MeRIP Kit (GenSeq Inc., China) following the manufacturer’s instructions. Both input samples without IP and m6A IP samples were used to generate the RNA-seq library using the NEBNext^®^ Ultra II Directional RNA Library Prep Kit (New England Biolabs, Inc., USA). The library quality was evaluated using the BioAnalyzer 2100 system (Agilent Technologies, Inc., USA). Library sequencing was performed on an Illumina NovaSeq 6000 instrument with 150 bp paired-end reads. Briefly, paired-end reads were harvested from the Illumina NovaSeq 6000 sequencer, and were quality controlled by Q30. After 3’ adaptor-trimming, low quality reads were removed using Cutadapt software (v1.9.3). Clean reads of all libraries were then aligned to the reference genome (MM10) using Hisat2 software (v2.0.4) (25). Methylated sites on RNAs (peaks) were identified by MACS software (26). Differentially methylated m6A sites (DMMSs) were identified by diffReps (27). Peaks identified by both software that had overlapping mRNA exons were determined and selected by home-made scripts. Using a fold change > 2 and P value < 0.00001, DMMSs were identified, and Gene Ontology (GO) and Kyoto Encyclopedia of Genes and Genomes (KEGG) analysis of overlapped differentially methylated genes was carried out. Visualization of DMMS alignment was performed through the Integrative Genomics Viewer (IGV, http://www.broadinstitute.org/igv/) according to the manufacturer’s instructions. We have uploaded the RNA-Seq data and MeRIP-Seq data in the GEO. The accession numbers are GSE184813 and GSE184814, respectively.

### Statistical Analysis

Data are expressed as mean ± SEM and were analyzed using Student’s t test in the SPSS Statistics v20.0. P values < 0.05 were considered to be statistically significant.

## Results

### General Characteristics of m6A Methylation in the Livers of HFrD and ND Mice

**Figure 1:**
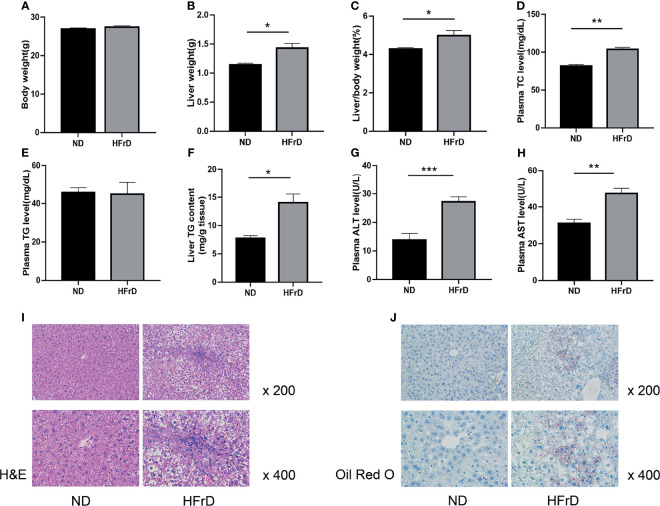
C57BL/6J mice fed a high-fructose diet for 2 weeks can induce NAFLD. **(A)** Body weight. **(B)** Liver weight. **(C)** Liver weight/body weight. **(D)** Plasma TC concentration. **(E)** Plasma TG concentration. **(F)** liver TG concentration. **(G)** Liver ALT concentration. **(H)** Liver AST concentration. **(I)** Liver HE staining and **(J)** Oil Red O staining. n=6 per group. *p < 0.05; **p < 0.01; ***p < 0.001.

We found that after feeding for 2 weeks, no significant changes in the weight of HFrD mice were observed compared to the control mice. However, the liver weight and liver weight/body weight ratio, as well as the liver TG levels were significantly increased in the HFrD group. Plasma TC, ALT, and AST levels were also significantly increased. The hepatic lipid accumulation was also confirmed by liver H&E and oil O red staining. Moreover, HFrD-induced liver steatosis and injury have also been observed in previous studies (28, 29). Therefore, our data demonstrated that we had successfully established a model of NAFLD in mice (**Figure 1**).

**Figure 2:**
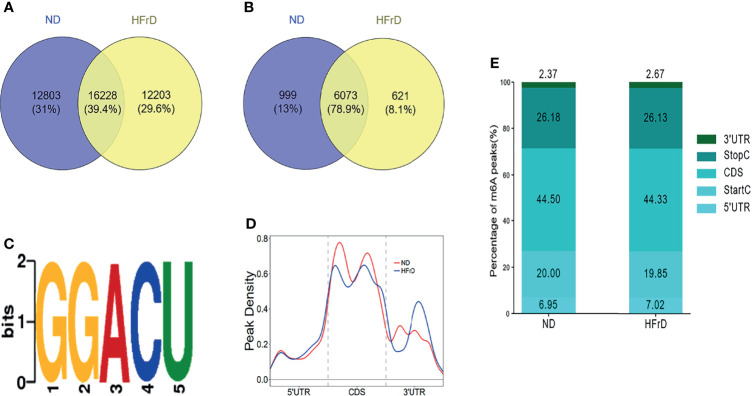
Overview of m6A methylation in the livers of HFrD and ND mice. **(A)** The overlap and difference of m6A peaks between HFrD and ND mice. **(B)** The overlap and difference of m6A modified genes between the two groups. **(C)** Compared with the control group, a common motif enriched in the m6A peak in the mRNA identified in the HFrD group. **(D)** The distribution of m6A peaks in the length of mRNA between HFrD and ND mice. **(E)** A stacked column chart showing the percentage of m6A peaks in five transcription fragments. The m6A peak is most enriched in the coding sequence fragments.

The liver tissues of HFrD and ND mice were analyzed by MeRIP-seq. The Venn diagram revealed that there were 16228 m6A peaks overlapping between the two groups. In addition, 12203 non-overlapping m6A peaks were observed in the HFrD mice. Finally, 6073 overlapping m6A-modified mRNAs were found in both groups, while 621 non-overlapping m6A-modified mRNAs were observed in the HFrD mice (**Figures 2A, B**)

We used DREME software (version: 4.12.10) to determine whether the m6A peaks identified in this study contained the RRACH sequence (R, purine; A, m6A; and H, non-guanine bases). Of the approximately 1000 m6A peaks analyzed, the GGACU motif sequence was found to be the most common. This strengthened the reliability of the m6A peaks, suggesting that m6A methylation plays an important role in NAFLD (**Figure 2C**). Next, the profile of the m6A peaks over the entire transcriptome was evaluated. Interestingly, we found that during the development of NAFLD, the m6A peaks were almost always distributed in the stop codon region, the coding sequence (CDS) and start codon region (**Figure 2D**).

In order to further investigate the specific location of m6A methylation on mRNA transcripts, the peaks were divided into five transcription fragments: 5’untranslated region (UTR); start codon fragment (400 nucleotides centered on the start codon); CDS; stop codon fragment (400 nucleotides centered on the stop codon); and 3’UTR. We found that the m6A peaks were mainly enriched in the CDS (44.33%–44.5%) and 3’UTR regions (26.13%–26.18%), consistent with previous studies (**Figure 2E**).

### Distribution and Biological Function of Differentially Methylated m6A Sites in HFrD and ND Mice

In total, 786 DMMSs were identified among m6A-modified mRNAs, of which 74.17% (583/786) were significantly up-regulated methylation sites (HFrD *vs* ND) under the threshold of |log2FC| >2 and p-value < 0.0001. **Tables 1** and **2** show the top ten up and down m6A methylation sites with the highest fold change in HFrD-induced NAFLD.

**Table 1:** Top ten hypermethylated peaks in HFrD induced NAFLD mice.

Chromosome	txStart	txEnd	GeneName	Foldchange
chr15	84112355	84112400	Pnpla5	288.8
chr9	99620199	99620460	A4gnt	145.8
chr2	129285621	129286100	Ckap2l	117.6
chr6	113758918	113759020	Atp2b2	100.5
chr7	45620161	45620500	Fut1	78.5
chr1	88247747	88247843	Mroh2a	70.7
chr19	4084877	4084908	Cabp2	58.9
chr7	46754021	46754314	Saa2	56.6
chr7	46751832	46751864	Saa2	54.9
chr6	113842641	113842645	Atp2b2	54.6

**Table 2:** Top ten hypomethylated peaks in HFrD induced NAFLD mice.

Chromosome	txStart	txEnd	GeneName	Foldchange
chr5	146833341	146833640	Rpl21	61.7
chr5	145694481	145694602	Cyp3a41a	50.3
chr5	145559081	145559217	Cyp3a41a	46.1
chr18	39373741	39373980	Arhgap26	24.1
chr16	22436201	22436288	Etv5	19.6
chr12	104266621	104266718	Serpina3i	19.2
chr17	26962913	26963087	Syngap1	13.7
chr8	48277666	48277680	Tenm3	12.5
chr7	92636821	92637142	Ankrd42	10.3
chr8	104839421	104839660	Ces2b	10.1

**Figure 3:**
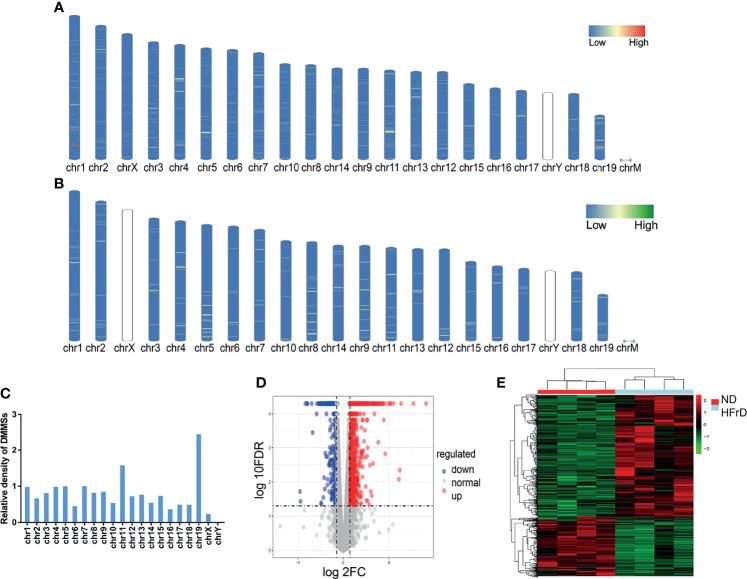
Distribution of differentially methylated m6A sites in HFrD and ND mice. The chromosome distribution of all hypermethylated m6A sites **(A)** and hypomethylated m6A sites **(B)** in mRNA between HFrD and ND mice. **(C)** The relative occupancy of different m6A methylation sites on each chromosome is normalized, according to the length of their respective chromosomes. **(D)** The volcano blot shows the distribution of differentially methylated m6A sites between HFrD and ND mice. **(E)** The heatmap shows the different m6A modification patterns between HFrD and ND mice.

All DMMSs in mRNA were located on chromosomes and their distribution maps were obtained. The four chromosomes with the most DMMSs were 1 (72), 11 (72), 4 (58), and 5 (57). However, when the number of DMMSs carried by chromosomes was normalized by the length of their respective chromosomes, the four chromosomes with the highest relative density of DMMSs were 19, 11, 7, and 5 (**Figures 3A–C**). The volcano plot and cluster analysis data revealed that there was a significant difference in the m6A methylation patterns between the HFrD and ND groups (**Figures 3D, E**).

**Figure 4:**
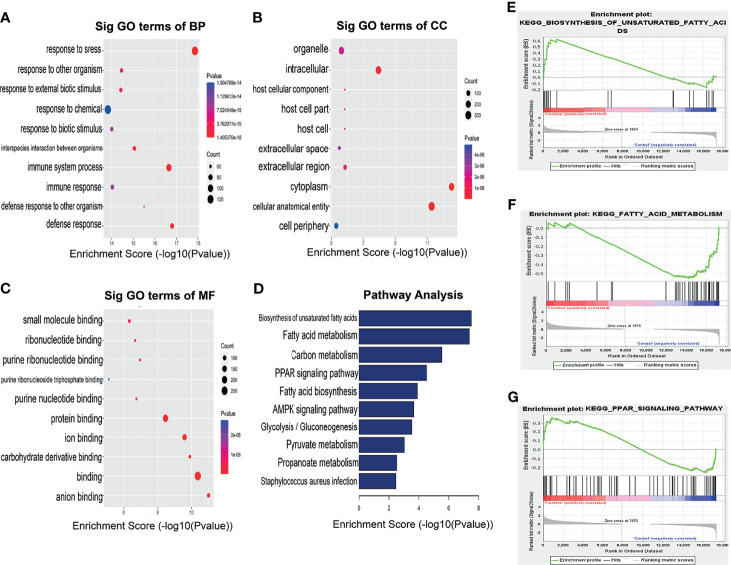
Biological function of differentially methylated m6A sites in HFrD and ND mice. GO analysis of differentially methylated m6A sites in **(A)** biological process (BP), **(B)** cellular component (CC), **(C)** molecular function (MF) categories. **(D)** KEGG analysis of differentially methylated m6A sites between HFrD and ND mice. **(E–G)** GSEA analysis of genes enriched in lipid metabolism pathways at m6A hypermethylation sites in HFrD mice.

In order to reveal the potential functions of genes encoding differential m6A sites in NAFLD, we selected these genes for functional enrichment analysis. In biological processes (BP), we found that mRNAs hypermethylated m6A sites were significantly enriched in the immune system processes. In cellular components (CC), these mRNAs were mainly enriched in the cytoplasm. In molecular functions (MF), these mRNAs were significantly associated with protein binding (**Figures 4A–C**).

KEGG pathway analysis and Gene Set Enrichment Analysis revealed that the genes with m6A hypermethylation sites in the HFrD group were enriched in lipid metabolism pathways, including unsaturated fatty acid synthesis, fatty acid metabolism and PPAR signaling pathways, indicating that m6A methylation is closely associated with these signaling pathways (**Figures 4D–G**).

### Comprehensive Analysis of MeRIP-Seq and RNA-Seq Data in HFrD and ND Mice

**Figure 5:**
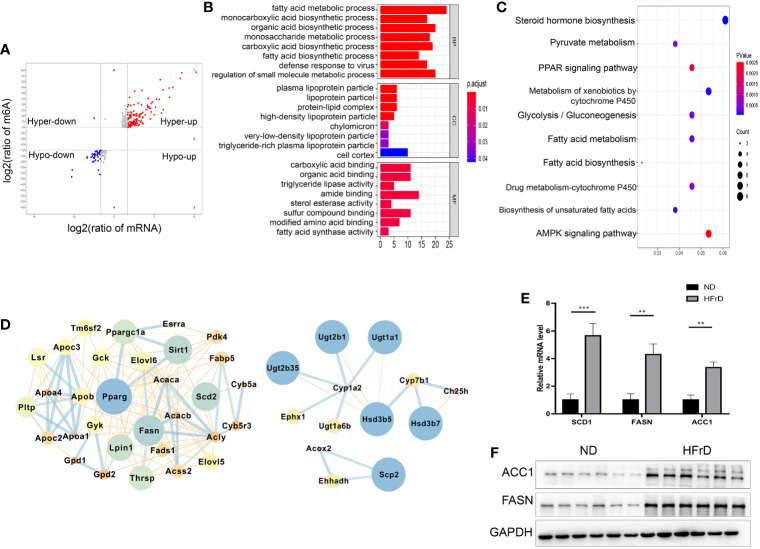
Comprehensive Analysis of MeRIP-Seq and RNA-Seq data in HFrD and ND mice. **(A)** The scatter plot shows the mRNA distribution with significant changes in m6A and mRNA levels between HFrD and ND mice. **(B)** GO analysis of mRNA with significant changes in m6A and mRNA levels between HFrD and ND mice. **(C)** KEGG analysis of mRNA with significant changes in m6A and mRNA levels between HFrD and ND mice. **(D)** Protein-protein interaction network of DMGs. According to the m6A modification and mRNA level common altered genes identified in HFrD and ND mice, conducted by Cytoscape software. The left represents m6A hypermethylation sites, and the right represents m6A hypomethylation sites. **(E)** Relative mRNA levels of Fasn, Scd1 and Acc1 in livers of HFrD and ND mice determined by qRT-PCR. **(F)** Protein levels of FASN and ACC1 proteins in livers of HFrD and ND mice determined by western blots. The data in **(E)** are presented as the means ± SEM. **p < 0.01; ***p < 0.001.

We combined MeRIP-seq and RNA-seq data and found that a total of 266 overlapping significantly DEGs had changes in both mRNA levels and m6A modification levels. Furthermore, of these 226 DEGs, 193 showed increases at the same time, indicating that these genes may be involved in the regulation of mRNA expression of m6A modified genes (**Figure 5A**). The top 20 genes whose m6A methylation levels and mRNA levels were both significantly changed in HFrD are listed in **Supplementary Table S1**. Interestingly, up-regulated mRNA and m6A modification levels were observed in more genes than down-regulation of mRNA and m6A modification levels. In addition, the fold change was higher, indicating that m6A modifications tend to be positively correlated with mRNA expression in NAFLD. Among these genes, we found that genes involved in fatty acid metabolism, including *FASN*, *Apoa4*, *Acaca*, and *Ppargc1a* were up-regulated. Consistent with these findings, subsequent enrichment analysis of KEGG and GO pathways also showed significant enrichment of fatty acid metabolic pathways, glycolysis/gluconeogenesis and pyruvate metabolism (**Figures 5B, C**). Cytoscape software was used to analyze the protein interaction network of those genes whose m6A methylation and mRNA levels were both significantly changed in HFrD-fed mice (30). *PPARγ*, *FASN*, *Lpin1*, and *SIRT1* were identified as core proteins (**Figure 5D**). The results of qRT-PCR and western blots further confirmed that expression levels of ACC1 and FASN were significantly increased in the HFrD group (**Figures 5E, F**). Therefore, these genes and their corresponding signaling pathways are of great significance to the protein interaction network and molecular events associated with NAFLD.

### Identification of Differentially Expressed Genes in Two NAFLD Models by RNA-Seq Data

**Figure 6:**
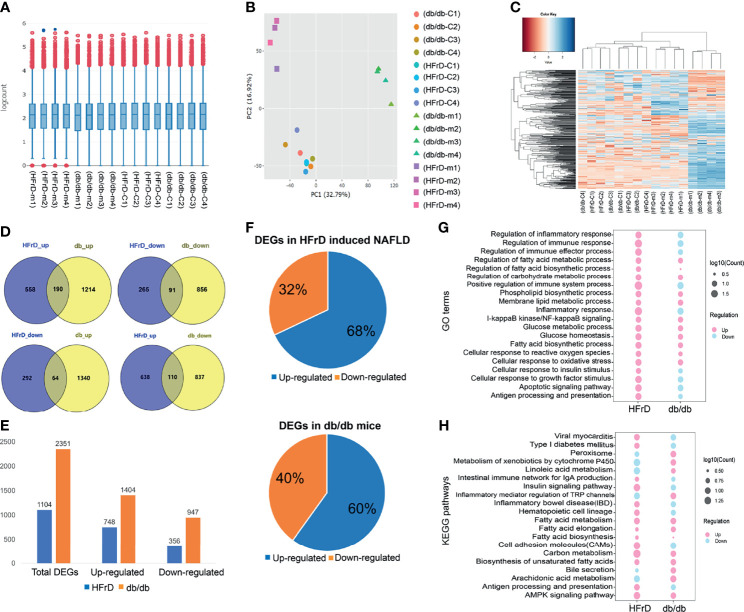
The liver gene expression patterns and differentially expressed genes (DEGs) of two different mouse NAFLD models were analyzed by RNA-Seq. **(A)** IQR box plot and **(B)** PCA analysis show that the read counts of each mouse sample are normalized after batch effect correction. **(C)** Heatmap shows the expression pattern between different models after batch effect correction. **(D)** The distribution of up-regulation and down-regulation DEGs detected in each model. **(E)** Quantification of DEGs in the two models. **(F)** Common up-regulated and down-regulated genes were detected in the HFrD and *db/db* groups. **(G)** GO analysis of common up-regulated and down-regulated DEGs between HFrD and *db/db* groups. **(H)** KEGG analysis of common up-regulated and down-regulated DEGs between HFrD and *db/db* groups.

Next, we compared different models of NAFLD. We performed a hierarchical cluster analysis on the liver gene expression of a HFrD-induced model of NAFLD and NAFLD caused by genetic defects (*db/db* mice). The normalized gene counts after batch effect adjustment were tested by IQR (interquartile range) box plots and principal correlation analysis, and it was confirmed that normalization was good, and that there was no significant bias in each group (**Figures 6A, B**). The normalized gene count was further calculated and plotted as a heat map (**Figure 6C**) showing two different gene expression patterns.

In order to identify the DEGs in each group, we used |logFC| > 1.5 and p < 0.05 as the threshold. We found significant differences in the expression of 1104 genes in the HFrD group. Compared with the control group, 748 genes (67.75%) were significantly up-regulated, and 356 genes (32.25%) were down-regulated. A total of 2351 DEGs were detected in the *db/db* group. Among them, 1404 genes (59.72%) were significantly up-regulated, while as many as 947 genes (40.28%) were down-regulated (**Figures 6D, E**).

We further analyzed the common DEGs of the two groups (**Figure 6F** and **Supplementary Table S2**) and found that 455 DEGs were changed in both the *db/db* mice and HFrD group. Some of these genes were related to lipid metabolism processes, such as fatty acid transporters and lipoprotein transporters (*FASN*, *CD36*, *Apoa4*, *Elovl5*, and *Abcd2*). **Supplementary Table S3** summarizes the top genes related to lipid metabolism in each group.

To further understand the common and different pathways associated with the distinct NAFLD models, we used the GO tool to compare DEGs from each group. As shown in **Figure 6G**, DEGs enriched in fatty acid metabolic processes, fatty acid biosynthetic processes, cellular responses to oxidative stress and glucose metabolic processes were significantly up-regulated in both *db/db* mice and HFrD mice. Interestingly, DEGs enriched in the regulation of immune processes and regulation of inflammatory responses were up-regulated in HFrD mice, but down-regulated in *db/db* mice.

We next used KEGG signal enrichment analysis, based on the identified DEGs, to study the pathways involved for each NAFLD model. The top 20 pathways identified in each group are listed in **Supplementary Tables S4, S5**. In the HFrD group, the up-regulated DEGs were mainly associated with fatty acid metabolism, the AMPK signaling pathway and the insulin signaling pathway, while the down-regulated DEGs were mainly related to metabolism of xenobiotics by cytochrome P450. In the *db/db* group, the up-regulated DEGs were mainly associated with fatty acid metabolism and the AMPK signaling pathway, while the down-regulated DEGs were mainly related to inflammatory bowel disease and the intestinal immune network for IgA production. These findings were consistent with the GO analysis data (**Figure 6H**).

### Analysis of RNA Binding Proteins (RBPs) and Integrative Genomics Viewer (IGV) of Differentially Methylated m6A Sites in Two NAFLD Models

**Figure 7:**
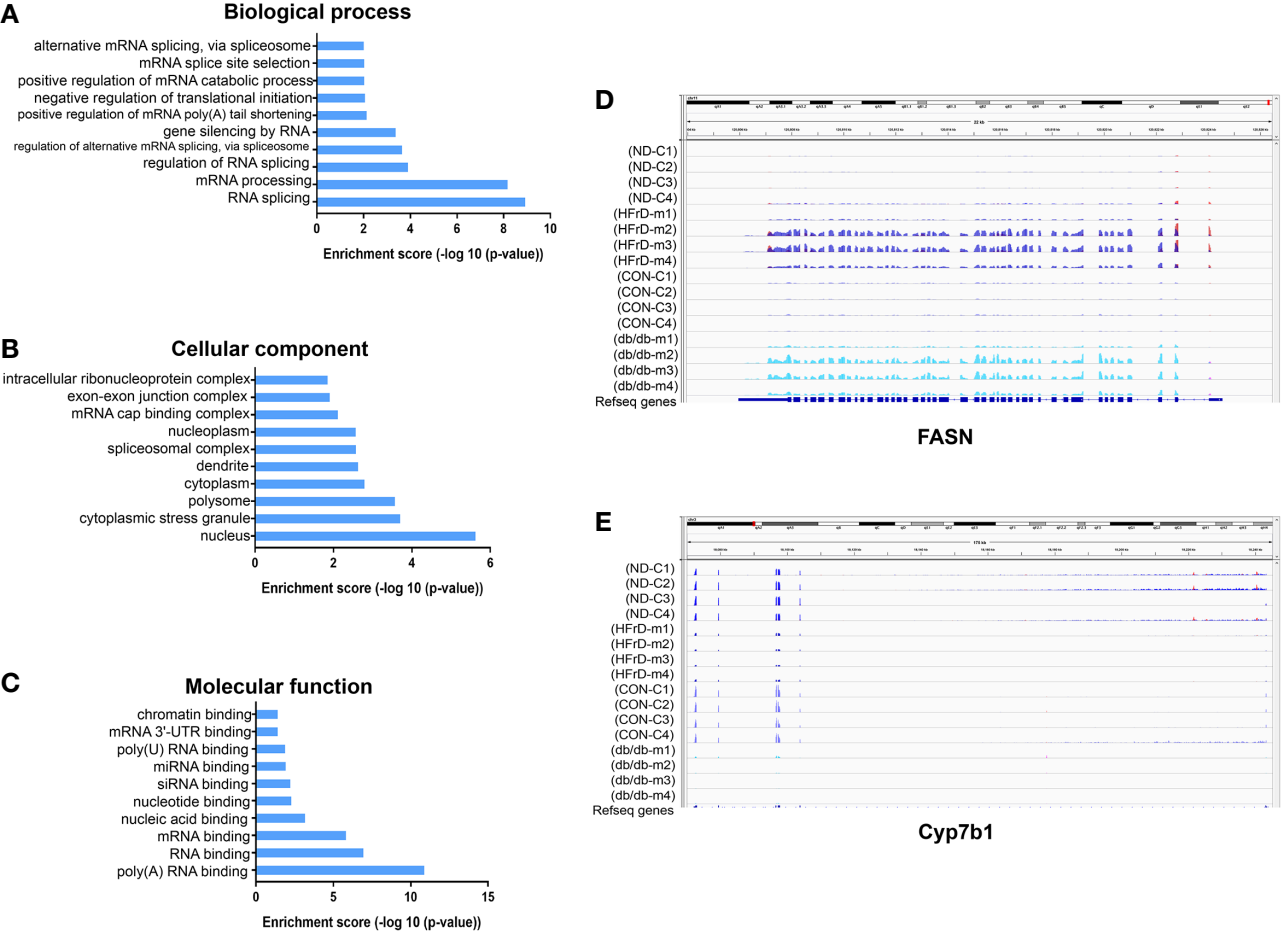
Analysis of RNA Binding Proteins (RBPs) and Integrative Genomics Viewer (IGV) of differentially methylated m6A sites in two NAFLD models. Identification of 23 RBPs binding to differentially methylated m6A sites. GO analysis shows the **(A)** biological process, **(B)**cellular component and **(C)** molecular function of the RBPs. The Integrative Genomics Viewer (IGV) shows the differentially methylated m6A sites in the HFrD and NC, as well as the *db/db* and control groups. **(D)** In HFrD and *db/db* mice, there is a representative hypermethylation gene compared to the control group. **(E)** In HFrD and *db/db* mice, there is a representative hypomethylation gene compared to the control group.

Multiple protein-coding genes containing DMMSs participate in many of the important processes and pathways described above. Therefore, we used the RMBase v2.0 database to explore the potential RBPs of the common DMMSs in the two NAFLD models (31). A total of 14 potential RBPs in the 28 common hypermethylation peaks and nine potential RBPs in the 11 common hypomethylation peaks were identified, indicating that a total of 16 proteins were predicted to be RBPs of 39 DMMSs. Next, these RBPs were analyzed for GO and KEGG enrichment. As shown in **Figures 7A–C**, in BP, these RBPs were mainly associated with RNA splicing and mRNA processing. In CC, these RBPs were mainly enriched in the nucleus. In MF, these RBPs were mainly enriched in RNA binding. The KEGG pathway data revealed that these RBPs were significantly associated with RNA transport and mRNA surveillance pathway in eukaryotes. These results indicate that RBPs play a key role in the regulation of gene expression and may be involved in m6A modification in NAFLD. We visualized the normalized m6A mapping results through IGV2.3, and determined the m6A patterns related to lipid metabolism in different NAFLD models. We found that m6A methylation of FASN was increased in both *db/db* and HFrD mice, while CYP7b1 was decreased in both groups. These findings were consistent with the intersection analysis data (**Figures 7D, E**).

## Discussion

In this study, we evaluated the role of m6A RNA methylation in mice fed a high fructose diet. First, we confirmed that short-term HFrD can induce liver steatosis, leading to the occurrence and development of NAFLD, which has been described previously (9, 28). Secondly, we found that in the HFrD mouse model, genes associated with m6A methylation were significantly involved in lipid metabolism-related pathways, and that the up-regulation of gene expression was often accompanied by up-regulation of m6A methylation. In addition, we also compared the similarities and differences between this HFrD-induced NAFLD model and NAFLD caused by genetic defects (*db/db* mice), and analyzed changes in m6A methylation modification in the two models.

m6A RNA methylation plays an important role in various endocrine tissues and organs (adipose tissue, pancreas islet and liver). Increasing evidence suggests that m6A methylation is involved in the process of adipogenesis. For example, FTO was shown to positively regulate autophagy and promote fat deposition in the pre-adipocytes of mice and pigs, whereas knocking out FTO negatively regulated autophagy while inhibiting lipid deposition (32). Mechanistically, FTO deficiency inhibits the expression of autophagy related 5 and autophagy related 7 by increasing mRNA m6A levels, leading to inactivation of autophagy and inhibiting adipogenesis (32). m6A is also involved in the biological functions of pancreatic β-cells. Rohit et al. found that the methyltransferases METTL3, ALKBH5, and YTHDF1 were down-regulated in diabetic pancreatic β-cells (33). Differentially methylated genes of m6A are enriched in insulin regulatory and diabetes-related metabolic pathways and several genes in the insulin/IGF1-AKT-PDX1 signal transduction pathway displayed reduced methylation levels. Furthermore, cell cycle arrest, increased basal insulin secretion, and impaired glucose-stimulated insulin secretion were observed in an *in vitro* endoC-βH1 β cell model and mouse β cell Mettl14 knockout animal model (33). m6A RNA methylation has been shown to play an important role in the process of hepatic steatosis. m6A RNA methylation and METTL3 levels were up-regulated in mice fed with high-fat diet for 16 weeks. Hepatocyte-specific METTL3 knockout reduced m6A methylation and total mRNA levels of the FASN gene, thereby inhibiting fatty acid metabolism (34). The FTO chemical inhibitor Entacapone was shown to reduce the weight and fasting blood glucose concentration of obese mice induced by diet by regulating the mRNA m6A modification of the Foxo1 gene (35). Previously, we demonstrated that in the liver tissue of *db/db* mice, the m6A modification and mRNA expression of lipid synthesis-related genes such as *Srebp-1c*, *FASN*, *Scd1*, *Acc1*, and *Gpat1* were significantly increased, while the expression of the m6A reader protein YT521-B homology domain containing 2 (YTHDC2) was significantly down-regulated (24). Inhibiting the expression of YTHDC2 in the liver of normal mice resulted in the deposition of TG; while overexpression of the YTHDC2 gene in the liver of obese mice improved liver lipid deposition and insulin resistance (24). In this study, we found that m6A modification patterns were different between HFrD and ND mice. Through the comprehensive analysis of RNA-seq and MeRIP-m6A-seq, m6A modifications tended to be positively correlated with mRNA expression in HFrD mice, meanwhile enrichment analysis revealed that these modifications were associated with the fatty acid metabolism and glycolysis pathways. Interestingly, we also found that these potential RBP genes of DMMSs were significantly enriched during RNA splicing, consistent with a previous study (36).

Since NAFLD is a heterogeneous disease with distinct pathogenic mechanisms, we used diet-induced (HFrD) and genetically defective (*db/db*) mouse models to study the differences and commonalities between them. Previous studies have found that excessive intake of fructose can lead to chronic low-grade inflammation, insulin resistance and obesity. Fructose induced expression of monocyte chemoattractant protein-1 and intracellular adhesion molecule-1, which led to the infiltration of macrophages into fat cells and subsequent inflammation (37). Fructose can modify intestinal metabolite levels, cause imbalance of intestinal flora, increase intestinal endotoxin translocation and plasma endotoxin levels, promote inflammation and mucosal barrier degradation, and is related to the loss of tight junction proteins in the duodenum (38). In addition, excessive fructose intake can inhibit the death of hypoxic villi cells in the intestine, which increased the length of intestinal villi and promoted the absorption of lipids (39). On the other hand, the *db/db* mouse has a natural mutation in the leptin receptor gene, rendering it nonfunctional. Therefore, these mice exhibit a phenotype similar to leptin-deficient *ob/ob* mice, and none of them spontaneously develop NASH unless stimulated further (40). Here, we found that lipid metabolism pathways were elevated in both HFrD and *db/db* mice, while immune processes and regulation of the inflammatory response were increased in HFrD mice and decreased in *db/db* mice. Thus, our findings confirm that differences in the NAFLD models exist depending on whether disease was induced by diet or genetic defects. In this study, we also found that m6A methylation of FASN was significantly increased in HFrD mice, while protein interaction network analysis revealed that FASN is the most central methylated protein of m6A in the disease process. These results indicate that FASN may be the key target of HFrD-induced NAFLD. In our study, Fragile X-linked Intellectual Disability Protein 1 (FMR1) was found to be the RBP gene of FASN. Recently, FMR1-KO mice were found to display a reduction in hepatic TG storage and smaller adipocytes with a reduced average surface, as compared to control animals (41). Combining the analysis of RBP genes by GO and KEGG analysis, we speculate that under the induction of dietary factors, these potential m6A-recognizing RBPs may regulate the alternative splicing, stabilization or translation of lipid metabolism-related genes, leading to lipid metabolism disorders. This hypothesis needs to be experimentally verified in future studies.

In summary, our study reveals that m6A RNA methylation plays an important role in the regulation of HFrD-induced NAFLD, which may add a novel insight into the molecular basis of NAFLD development.

## Data Availability Statement

The datasets presented in this study can be found in online repositories. The names of the repository/repositories and accession number(s) can be found below: https://www.ncbi.nlm.nih.gov/geo/, accession IDs: GSE184813 and GSE184814.

## Ethics Statement

The animal study was reviewed and approved by Animal Care Committee of Shanghai Jiao Tong University School of Medicine.

## Author Contributions

YLu and YP designed and directed the study. YLuo, LX, and BZ performed animal experiments. YLuo, ZZ, and LX performed multi-omic analysis. XW, YLin, XD, and FL contributed to the discussion. All authors contributed to the article and approved the submitted version.

## Funding

This study was supported by the National Key Research and Development Program of China (No. 2018YFA0800402), Program of Shanghai Academic/Technology Research Leader by Shanghai Municipal Science and Technology Committee (No. 21XD1423400), the National Natural Science Foundation of China (Nos. 81970751, 81870596, 81870594, and 81820108008), Shanghai Jiao Tong University Research Funding on Medical, Engineering Interdisciplinary Project (YG2019GD05).

## Conflict of Interest

The authors declare that the research was conducted in the absence of any commercial or financial relationships that could be construed as a potential conflict of interest.

## Publisher’s Note

All claims expressed in this article are solely those of the authors and do not necessarily represent those of their affiliated organizations, or those of the publisher, the editors and the reviewers. Any product that may be evaluated in this article, or claim that may be made by its manufacturer, is not guaranteed or endorsed by the publisher.
