# Supplementary material for: Analysis of N6-Methyladenosine Methylation Modification in Fructose-Induced Non-Alcoholic Fatty Liver Disease
Source: Front Endocrinol (Lausanne). 2021 Dec 7;12:780617. doi: 10.3389/fendo.2021.780617 (PMC8688819; doi:10.3389/fendo.2021.780617)
Supplement: Supplementary file 1 [file Table_1.docx]

**Supplementary Table S1. List of top 20 genes that exhibit a significant change in both m6A level and mRNA transcript abundance in HFrD induced NAFLD compared with normal mice**

| **GeneName** | **Pattern** | **Chorm** | **m6A level change** | | | | **mRNA level change** | | |
| --- | --- | --- | --- | --- | --- | --- | --- | --- | --- |
|  |  |  | **Peak start** | **Peak end** | **Fold change** | **p-value** | **strand** | **Fold change** | **p-value** |
| Atp2b2 | Hyper-up | chr6 | 113758918 | 113759020 | 100.5 | 2.62E-09 | - | 33.55324 | 5.00E-05 |
| Saa2 | Hyper-up | chr7 | 46754021 | 46754314 | 56.5621 | 1.66E-06 | + | 71.26145 | 5.00E-05 |
| Chrna4 | Hyper-up | chr2 | 181022310 | 181022340 | 38.8 | 2.11E-05 | - | 30.92861 | 0.00455 |
| Ppargc1a | Hyper-up | chr5 | 51553801 | 51553921 | 38.5 | 7.93E-06 | - | 2.306827 | 0.00745 |
| Pdzk1ip1 | Hyper-up | chr4 | 115093801 | 115093899 | 36 | 1.13E-05 | + | 10.96771 | 5.00E-05 |
| Saa1 | Hyper-up | chr7 | 46740498 | 46740580 | 27.22997 | 1.02E-06 | - | 17.41478 | 5.00E-05 |
| Apoa4 | Hyper-up | chr9 | 46243261 | 46243459 | 22.62093 | 1.33E-06 | + | 21.04871 | 5.00E-05 |
| Fabp5 | Hyper-up | chr3 | 10012547 | 10012718 | 19.26353 | 3.62E-07 | + | 12.71174 | 5.00E-05 |
| Cdh18 | Hyper-up | chr15 | 23474101 | 23474320 | 18.73077 | 3.3E-06 | + | 4.241193 | 0.00175 |
| Abcd2 | Hyper-up | chr15 | 91191661 | 91191807 | 17.96154 | 4.08E-05 | - | 2.905024 | 5.00E-05 |
| Ces2b | Hypo-down | chr8 | 104839421 | 104839660 | 10.13514 | 6.44E-08 | + | -9.03078 | 5.00E-05 |
| Raet1d | Hypo-down | chr10 | 22361893 | 22361909 | 8.77451 | 1.62E-05 | + | -2.09062 | 0.00235 |
| Selenbp2 | Hypo-down | chr3 | 94704301 | 94704413 | 8.358162 | 7.61E-11 | + | -8.79723 | 5.00E-05 |
| Serpinb1a | Hypo-down | chr13 | 32842161 | 32842580 | 6.729885 | 3.82E-06 | - | -2.23225 | 0.0025 |
| Slc25a48 | Hypo-down | chr3 | 94693555 | 94693660 | 6.683398 | 5.32E-12 | + | -8.79723 | 5.00E-05 |
| Asap3 | Hypo-down | chr13 | 56465061 | 56465088 | 5.107011 | 1.23E-07 | + | -1.57821 | 0.0006 |
| Cd276 | Hypo-down | chr4 | 136236374 | 136236420 | 4.596685 | 1.36E-05 | + | -1.63093 | 0.00305 |
| Tsku | Hypo-down | chr4 | 107068543 | 107068680 | 3.929688 | 1.26E-06 | + | -1.92751 | 0.01945 |
| Pcp4l1 | Hypo-down | chr7 | 98361277 | 98361328 | 3.92126 | 1.31E-07 | - | -2.83974 | 5.00E-05 |
| Lifr | Hypo-down | chr1 | 171173621 | 171173920 | 3.803661 | 4.69E-07 | - | -2.29051 | 0.00315 |
