# Supplementary material for: Analysis of N6-Methyladenosine Methylation Modification in Fructose-Induced Non-Alcoholic Fatty Liver Disease
Source: Front Endocrinol (Lausanne). 2021 Dec 7;12:780617. doi: 10.3389/fendo.2021.780617 (PMC8688819; doi:10.3389/fendo.2021.780617)
Supplement: Supplementary file 2 [file Table_2.docx]

**Supplementary Table S2. Common key DEGs in each mouse model of NAFLD in comparison with each other**

| **Types of DEGs** | **Gene Names** |
| --- | --- |
| DEGs upregulated in both HFrD induced NAFLD and db/db mice (n=190) | Abca3，Abcd2，Acaca，Acacb，Acer2，Acly，Acnat2，Acsl5，Apoa4，Cd36，Cyp4a14，Cyp4a31，Elovl5，Elovl6，Fabp4，Fasn，Gpam，Insig2，Lipg，Mfsd2a，Pdk4，Pnpla3，Ppargc1a，Scd2，Smpd3，Thrsp，Unc119，1810011O10Rik，4930452B06Rik，2010003K11Rik，2010012O05Rik，2510049J12Rik，A4gnt，Abca3，Acpp，Acss3，Adck3，Ano10，Anxa2，Anxa5，Aqp8，Arhgap10，Arntl，As3mt，BC052040，Cdcp1，Cldn2，Cyp17a1，Cyp2c38，D130043K22Rik，Defb1，Dntt，Dtx4，Ebpl，Enpep，Extl1，Fam124a，Fam198a，Fam84b，Fmo4，Fst，G6pc，Gale，Gas6，Gm5549，Gpd2，Gtdc1，Hamp2，Hcls1，Ifi27l2b，Lcn2，Lgals1，Lgalsl，Lhx6，Lrfn3，Lrtm1，Maged2，Me1，Mmd2，Morc4，Mr1，Mt1，Mt2，Olfm3，Pde4d，Pde7b，Pdk1，Pgd，Pgrmc2，Pik3c2g，Pklr，Pls1，Ppp1r3b，Prob1，Pstpip2，Ptp4a3，Rasgrp2，Rdh16，Rnd2，Robo1，S100a10，S100a11，Saa1，Saa2，Samd9l，Serpina7，Slc16a5，Slc17a4，Sntb1，St5，Stap1，Sult1e1，Synj2，Tbc1d31，Tceal8，Them6，Tlr7，Tmem71，Tmie，Tnfaip3，Tnfrsf11b，Tor3a，Trim7，Tspyl4，Tubb2a，Ugt1a5，Vat1，Wfdc2，Zfp385a |
| DEGs downregulated in both HFrD induced NAFLD and db/db mice (n=91) | 8430419L09Rik，Abca8a，Adh6-ps1，Aox3，Atxn1，Bcl6，Btg3，C8b，Capn8，Cd276，Ces1b，Ces2a，Ces2b，Ces3a，Ces3b，Ces4a，Clec2d，Csad，Cyp1a2，Cyp2c70，Cyp7b1，Dact1，E130012A19Rik，Enho，F11，Fbxo21，Foxq1，Gm3839，Gstp1，Hamp，Hsd17b6，Hsd3b5，Idi1，Igfals，Igfbp2，Igj，Irf5，Keg1，Lama3，Lifr，Mcm10，Mup1，Mup11，Mup12，Mup14，Mup15，Mup17，Mup2，Mup7，Mup10，Nrep，Phlda1，Rapgef4，Rbbp4，Scnn1a，Selenbp2，Serpina1e，Serpine2，Smad9，Sntg2，Sort1，Spsb4，Tspan33，Tstd1，Ttc39c |
| DEGs upregulated in HFrD induced NAFLD but downregulated in db/db mice (n=110) | 1600029D21Rik，Apobec3，Arl4d，Arpc1b，Arrb2，Bcmo1，Bst2，Camk2b，Cd300lf，Cdpf1，Chic2，Col5a3，Coro1a，Cxcl13，Eif4ebp1，Elovl2，Emr4，Fabp5，Fam46a，Gadd45g，Gbp2b，H2-Aa，H2-Ab1，H2-Dma，H2-DMb1，H2-Eb1，H2-Q7，Hapln4，Hgfac，Hk3，Ifitm3，Igfbp1，Igsf8，Il1b，Laptm5，Lpin1，Lst1，Ltbp1，Marco，Metrnl，Pdzk1ip1，Pilra，Pirb，Pld4，Ppp1r3g，Pydc4，Rps28，Rrm2，Rtp4，Slc3a1，Slc6a8，Smoc2，Syne4，Tff3 |
| DEGs upregulated in db/db but downregulated in HFrD induced NAFLD mice(n=64) | 2610305D13Rik，4931406C07Rik，9130409I23Rik，Abat，Acot1，Aldh1a1，Aldh3a2，Aspa，Chpt1，Chrna2，Cyp26a1，Cyp2b10，Cyp2c29，Cyp2c40，Cyp2c69，Cyp3a59，E2f3，Ephx1，Fmo5，Gm14325，Gm14327，Grk4，Gsta2，Ildr2，Lrit1，Nat8，Obp2a，Pm20d1，Ppp4r4，Raet1d，S1pr5，Serpinb1a，Sult1b1，Tlr5，Ugt3a1 |
