# Supplementary material for: Analysis of N6-Methyladenosine Methylation Modification in Fructose-Induced Non-Alcoholic Fatty Liver Disease
Source: Front Endocrinol (Lausanne). 2021 Dec 7;12:780617. doi: 10.3389/fendo.2021.780617 (PMC8688819; doi:10.3389/fendo.2021.780617)
Supplement: Supplementary file 3 [file Table_3.docx]

**Supplementary Table S3. Key DEGs significantly changed involved in lipid metabolic process in the liver of each mouse model.**

| **HFrD induced NAFLD** | | | **db/db** | | |
| --- | --- | --- | --- | --- | --- |
| Gene name | log2(fold change) | FDR | Gene name | log2(fold change) | FDR |
| Pnpla3 | 7.25963 | 0.00103855 | Pnpla3 | 4.94022 | 0.000578 |
| Apoa4 | 4.39566 | 0.00103855 | Acnat2 | 4.83398 | 0.000578 |
| Fabp5 | 3.66809 | 0.00103855 | Mogat1 | 4.55085 | 0.000578 |
| Elovl6 | 3.16709 | 0.00103855 | Mogat2 | 4.53059 | 0.000578 |
| Acly | 3.08555 | 0.00103855 | Cd36 | 4.45396 | 0.000578 |
| Acacb | 2.9914 | 0.00103855 | Atp8b5 | 3.59469 | 0.000578 |
| Fasn | 2.7762 | 0.00103855 | Vldlr | 3.57999 | 0.000578 |
| Elovl5 | 2.72147 | 0.00103855 | Smpd3 | 3.17173 | 0.002417 |
| Acaca | 2.51125 | 0.00103855 | Elovl6 | 3.11782 | 0.000578 |
| Apoc2 | 1.54845 | 0.00103855 | Scd1 | 3.04407 | 0.000578 |
| Fabp4 | 1.53441 | 0.00103855 | Elovl5 | 2.46632 | 0.000578 |
| Lpin1 | 1.46698 | 0.00103855 | Gpam | 2.4615 | 0.000578 |
| Insig2 | 1.20968 | 0.00103855 | Insig2 | 2.36451 | 0.000578 |
| Fads2 | 1.16528 | 0.00103855 | Pparg | 2.28283 | 0.000578 |
| Acsl5 | 1.09119 | 0.00103855 | Acot11 | 2.10149 | 0.000578 |
| Cd36 | 1.08641 | 0.00103855 | Fasn | 2.07406 | 0.000578 |
| Smpd3 | 1.07576 | 0.0801825 | Pebp1 | 1.96733 | 0.000578 |
| Gpam | 2.23793 | 0.00103855 | Thrsp | 1.93499 | 0.000578 |
| Abcd2 | 1.53855 | 0.00103855 | Abca3 | 1.84776 | 0.000578 |
| Ppargc1a | 1.20591 | 0.0637676 | Abhd2 | 1.72915 | 0.000578 |
| Cyp7b1 | -2.01936 | 0.00103855 | Slc27a3 | 1.66773 | 0.04015 |
| Acot1 | -1.5879 | 0.00103855 | Scd2 | 1.66453 | 0.000578 |
| Cyp1a2 | -1.37627 | 0.00103855 | Apoa4 | 1.64582 | 0.000578 |
|  |  |  | Acaa1b | 1.5756 | 0.000578 |
|  |  |  | Acly | 1.45867 | 0.000578 |
