# Supplementary material for: Analysis of N6-Methyladenosine Methylation Modification in Fructose-Induced Non-Alcoholic Fatty Liver Disease
Source: Front Endocrinol (Lausanne). 2021 Dec 7;12:780617. doi: 10.3389/fendo.2021.780617 (PMC8688819; doi:10.3389/fendo.2021.780617)
Supplement: Supplementary file 4 [file Table_4.docx]

**Supplementary Table S4. Top 20 overrepresented pathways of KEGG analysis of DEGs in HFrD group (according to P value).**

| **Pathway ID** | **Definition** | **Fisher-P value** | **Selection Counts** |
| --- | --- | --- | --- |
| **UP-regulated** | | | |
| mmu04152 | AMPK signaling pathway | 0.000621 | 15 |
| mmu04612 | Antigen processing and presentation | 0.010518 | 10 |
| mmu01040 | Biosynthesis of unsaturated fatty acids | 0.000139 | 7 |
| mmu01200 | Carbon metabolism | 8.90E-13 | 27 |
| mmu04514 | Cell adhesion molecules (CAMs) | 0.00516 | 16 |
| mmu00061 | Fatty acid biosynthesis | 0.002775 | 4 |
| mmu00062 | Fatty acid elongation | 0.027152 | 4 |
| mmu01212 | Fatty acid metabolism | 1.19E-05 | 11 |
| mmu04640 | Hematopoietic cell lineage | 0.014446 | 9 |
| mmu05321 | Inflammatory bowel disease (IBD) | 0.001135 | 9 |
| mmu04910 | Insulin signaling pathway | 0.000543 | 16 |
| mmu04672 | Intestinal immune network for IgA production | 0.041883 | 5 |
| mmu04940 | Type I diabetes mellitus | 0.019902 | 8 |
| mmu05416 | Viral myocarditis | 0.009119 | 10 |
| **DOWN-regulated** | | | |
| mmu00590 | Arachidonic acid metabolism | 1.42E-07 | 12 |
| mmu04976 | Bile secretion | 0.049128 | 4 |
| mmu04750 | Inflammatory mediator regulation of TRP channels | 3.14E-05 | 11 |
| mmu00591 | Linoleic acid metabolism | 3.14E-13 | 14 |
| mmu00980 | Metabolism of xenobiotics by cytochrome P450 | 5.46E-14 | 16 |
| mmu04146 | Peroxisome | 0.005423 | 6 |
