# Supplementary material for: Analysis of N6-Methyladenosine Methylation Modification in Fructose-Induced Non-Alcoholic Fatty Liver Disease
Source: Front Endocrinol (Lausanne). 2021 Dec 7;12:780617. doi: 10.3389/fendo.2021.780617 (PMC8688819; doi:10.3389/fendo.2021.780617)
Supplement: Supplementary file 5 [file Table_5.docx]

**Supplementary Table S5. Top 20 overrepresented pathways of KEGG analysis of DEGs in db/db group (according to P value).**

| **Pathway ID** | **Definition** | **Fisher-P value** | **Selection Counts** |
| --- | --- | --- | --- |
| **UP-regulated** | | | |
| mmu04152 | AMPK signaling pathway | 0.0343868 | 9 |
| mmu00590 | Arachidonic acid metabolism | 5.64E-05 | 12 |
| mmu04976 | Bile secretion | 0.0007024 | 9 |
| mmu01040 | Biosynthesis of unsaturated fatty acids | 1.68E-07 | 9 |
| mmu01200 | Carbon metabolism | 0.0197401 | 9 |
| mmu00061 | Fatty acid biosynthesis | 0.011207 | 3 |
| mmu00062 | Fatty acid elongation | 2.04E-05 | 7 |
| mmu01212 | Fatty acid metabolism | 7.53E-06 | 10 |
| mmu04750 | Inflammatory mediator regulation of TRP channels | 0.0117239 | 10 |
| mmu00591 | Linoleic acid metabolism | 0.0071554 | 6 |
| mmu00980 | Metabolism of xenobiotics by cytochrome P450 | 0.0003612 | 9 |
| mmu04146 | Peroxisome | 0.0005409 | 10 |
| **DOWN-regulated** | | | |
| mmu04612 | Antigen processing and presentation | 0.0096983 | 7 |
| mmu04514 | Cell adhesion molecules (CAMs) | 0.0041431 | 11 |
| mmu04640 | Hematopoietic cell lineage | 0.0053758 | 7 |
| mmu05321 | Inflammatory bowel disease (IBD) | 9.39E-05 | 8 |
| mmu04910 | Insulin signaling pathway | 0.0239779 | 8 |
| mmu04672 | Intestinal immune network for IgA production | 0.0040178 | 5 |
| mmu04940 | Type I diabetes mellitus | 0.0005572 | 8 |
| mmu05416 | Viral myocarditis | 0.0005144 | 9 |
